# A Medical Causerie

**Published:** 1915-06-12

**Authors:** 


### A MEDICAL CAUSERIE.

The fund raised for the relief of the Belgian doctors and pharmacists has made a wide appeal to members of the medical and pharmaceutical professions both in the United Kingdom and in other parts of the Empire. It is understood that a considerable sum has been subscribed, and contributions still continue to arrive. A noteworthy feature among recent donations is a sum of over £300 which has been received from thirty of the Sunderland practitioners, this sum representing the fees paid to these gentlemen for the examination of recruits for the Army. It is seemly that so generous a gift should receive a special acknowledgment. From communications received by the British committee through the organisation which controls the distribution of the money in Brussels, some notion may be gained of the disasters and losses which have fallen on our Belgian colleagues. Such help as money can give is still essential, even if the object is merely to secure the bare necessaries of life for some of those who, prior to the war, enjoyed comfortable incomes and successful practices. The honorary treasurer of the fund is Dr. H. A. Des Vœux, and subscriptions should be sent to him at 14 Buckingham Gate, S.W.

The decision of the Medical Defence Union to raise as an issue in a Court of Law the surcharging of medical practitioners against whom "excessive prescribing" is alleged, is creating much interest. There is no doubt that a very bitter feeling is growing up in connection with this matter, and, indeed, hardly anything can be more obnoxious to a medical man than a proposal to survey, by means of a committee, his methods of treatment. It may be quite right that the need for economy should be emphasised, and this could readily be effected by distributing to the practitioner concerned information showing the cost of his prescriptions in relation to the average charge. But that men are to be summoned before a committee to be cross-questioned on their prescribing habits is really more than flesh and blood can stand. In some quarters it is being urged that in existing circumstances contention is to be avoided, and that it is better to suffer wrong than to encourage strife. The answer is the one appropriate to those who advocate the abolition of capital punishment, "*Que Messieurs les assassins commencent.*"

The arrangements at the new King George Hospital have come in for criticism in several respects. It has been urged, for example, that the appointment of a large resident staff of young men means the occupation at home of just the type of doctor who is wanted at the Front. The answer to this is that the hospital expects to have to deal with numbers of severely wounded men, and that, in view of the contingencies which may arise at any moment, it would be impossible to rely on the help of outside practitioners, who might or might not be available when required. Whether the possibility of utilising the services of fifth-year medical students was considered does not appear. Such an arrangement would have set free the qualified men for active service, and, if work at the hospital had been allowed to count as part of the curriculum (and surely, in the circumstances, this ought to have been possible), the students would have been moving towards graduation, and thus to readiness for joining the Army in their turn. For the present, the decision which has been taken must be accepted, but its effects ought to be closely watched. In any event, the appointments ought to be for a limited period. They should be regarded not as comfortable home billets for a limited number of men, but rather as opportunities for training for the duties of the field. The nomination of the whole of the staff, visiting as well as resident, seems to have been in the hands of two surgeons, and now that this is known, some of the elections, which hitherto had excited surprise, are no longer a cause of wonder. One of the two grand electors was largely responsible for the election some years ago of the staff of the newly-constituted Hampstead Hospital, and his personal experiences in this adventure might well have taught him that his capacities were better suited for other fields.

Attention has previously been drawn in this column to the hard lot of the Territorial medical officer who, on mobilisation, was compelled to join his unit, even though this was stationed at a place far removed from his area of practice. Obviously, in such circumstances, his professional income is largely destroyed, while his military pay, even if he has several years' service behind him, is hardly more than £300 to £400 per annum. Add to this the consideration that a newly-joined lieutenant in the R.A.M.C. at once draws a salary of more than £400, and there is no wonder that the sense of injustice is acute. Mr. Arnold Bennett deserves the thanks not only of the officers concerned but of the whole profession for his recent article in the *Westminster Gazette*. It is to be hoped that this public notice will lead the authorities to recognise the unfairness of the existing arrangements; these inflict a very much heavier burden on doctors who have given time and interest for years to the success of the Territorial Forces than that placed on the shoulders of those who did nothing in this direction prior to the outbreak of the war. This is not to say that those who have arrived at the "eleventh hour" are treated with undue generosity, but it is a plea for those who have borne "the burden and heat of the day." Even in the parable the latter were not, as is the case here, paid *less* than those who appeared late in the day.

The Birthday Honours List is not quite unmindful of the peaceful activities of the country. Dr. James Mackenzie's patient and interesting work on cardiac diseases has had a long struggle to secure recognition, and the knighthood conferred on him will be generally approved. Some few months ago we had occasion to congratulate him on his election as a Fellow of the Royal Society, and we now applaud his appearance as a Knight Bachelor. The List is dealt with fully in our Notes this week.

